# One year after partial legalisation in Germany: Trends in cannabis use among young people between 2008 and 2025

**DOI:** 10.25646/14268

**Published:** 2026-07-01

**Authors:** Boris Orth, Stephanie Eckhardt, Anika Nitzsche

**Affiliations:** 1 Federal Institute of Public Health, Unit Q 3 – Evaluation, Methods, Research Data, Cologne, Germany; 2 Federal Institute of Public Health, Unit T 4 – Addiction Prevention, Cologne, Germany

**Keywords:** Cannabis, Cannabis use, Partial legalisation, Prevalence, Temporal trends, Adolescents, Young adults, Germany

## Abstract

**Background:**

One year after the partial legalisation of cannabis, the effects on cannabis use among young people in Germany were investigated.

**Methods:**

Based on twelve representative studies from 2008 to 2025, changes in the 12-month prevalence of cannabis use among 12- to 17-year-old adolescents and 18- to 25-year-old young adults were presented, and trends over time were estimated.

**Results:**

Among female and male adolescents as well as young women, there was no statistically significant change in the 12-month prevalence of cannabis use between 2023 and 2025. In contrast, a statistically significant increase in consumption was observed among young men. However, this does not represent a new development but rather continues a trend that has been observed in this group since 2008.

**Conclusions:**

One year after partial legalisation, there was no evidence of an immediate effect on the prevalence of cannabis use among young people in Germany. Further studies should monitor future developments.

**Key messages**

► Between 2023 and 2025, the 12-month prevalence of cannabis use remained unchanged among female and male adolescents and young women.
► In 2025, 4.6 % of female adolescents and 7.2 % of male adolescents reported having used cannabis. Among young adults, the 12-month prevalence rates are notably higher, at 18.8 % (women) and 31.6 % (men).
► Young men showed a higher 12-month prevalence of cannabis use in 2025 than in 2023. However, this does not represent a new development but rather continues a rising trend that has been observed since 2008.
► Overall, there are no indications of any short-term effects of partial legalisation on the prevalence of cannabis use among young people.

## 1. Introduction

Regular and long-term cannabis use is associated with various adverse health effects – particularly among young people – including, for example, respiratory and cardiovascular diseases, gastrointestinal complaints, addiction, psychosis, mood and anxiety disorders, and suicidal behaviour (for an overview, see [1]). The prevalence of cannabis use among young people is therefore a matter of great importance for public health. Following the partial legalisation of cannabis under the Consumer Cannabis Act (KCanG) of 27 March 2024 [2], the question therefore arises as to whether cannabis use is increasing among adolescents and young adults in Germany. International studies examining changes in cannabis use following legalisation (e.g. in Canada and nearly half of the US states) have predominantly found increases in both prevalence and frequency of use among young adults, whereas findings for adolescents remain inconsistent [3–6]. Furthermore, an increase in cannabis-related emergencies (e.g. acute intoxication, psychosis) has been observed in these countries [7, 8].

In Germany, the Federal Institute of Public Health (BIÖG)^1^ regularly conducts representative surveys on cannabis use among adolescents and young adults. A previous article in issue 3/2025 of the Journal of Health Monitoring examined trends in these age groups from 2008 to 2023 [9], i.e. up to one year before the partial legalisation of cannabis. The BIÖG now has new data from the 2025 Drug Affinity Study [10]. This article aims to use this data to extend the previously published trend analyses up to the year 2025, i.e. one year after the partial legalisation came into force. Firstly, the focus will be on short-term changes in the 12-month prevalence of cannabis use between 2023 and 2025. Secondly, these current developments will be contextualised within the longer-term trends from 2008 to 2025. Finally, initial conclusions will be drawn and discussed regarding the potential impact of the partial legalisation of cannabis on young people´s consumption behaviour in Germany.

## 2. Methods

For the 2025 Drug Affinity Study, a total of 7,001 adolescents and young adults aged between 12 and 25 were surveyed via computer-assisted telephone interviews (CATI) between 22 April and 3 July 2025. Apart from minor adjustments to the content of the interview that did not affect prevalence of use, the methodological approach corresponded exactly to that of the last Drug Affinity Study from 2023 [11]. The study was conducted using a dual-frame approach [12], with the mix of landline and mobile phone samples set at 60 % to 40 %.

Forsa, Gesellschaft für Sozialforschung und statistische Analysen mbH, was commissioned to carry out the sampling based on the telephone sampling system of the Arbeitskreis Deutscher Markt- und Sozialforschungsinstitute, ADM and to collect the data. In 2025, the response rates were 35.5 % for the landline sample and 27.4 % for the mobile phone sample. The differing probabilities of respondents being selected resulting from the sampling design were balanced out by design weighting. Adjustment weighting aligned the sample distributions regarding gender, age, education and region with those of the 12- to 25-year-old population in Germany.

The analysis of trends from 2008 to 2025 was conducted in the same way as our trend analyses for the period 2008 to 2023, which have already been described in detail elsewhere [9]: Using logistic regression analyses with the survey year as a continuous independent variable, temporal trends from 2008 to 2025 were modelled and examined to determine how the prevalence of cannabis use among female and male adolescents and young adults has changed. For this paper, additional logistic regression analyses were calculated in which the survey year was modelled as a categorical variable (reference year 2025). This enables us to conclude which of the preceding years differed significantly from 2025 and to make a direct comparison between 2023 and 2025 – that is, the prevalence of cannabis use one year before and one year after partial legalisation.

The 12-month prevalence of cannabis use represents the percentage of respondents who have used cannabis in the past 12 months. To this end, information on the frequency of use over the past twelve months (‘not at all’, ‘once’, ‘twice’, ‘three to ten times’ or ‘more often’) was summarised into a dichotomous variable (‘no use’, ‘use’). Gender was recorded using the question ‘Are you male or female?’. From 2021 onwards, interviewers could also select the category ‘diverse’ if respondents so wished.

## 3. Results

A total of 3,031 adolescents aged 12 to 17 and 3,970 young adults aged 18 to 25 took part in the 2025 Drug Affinity Study. One adolescent and one young adult did not provide any information regarding cannabis use in the past twelve months. Both were excluded from the trend analyses. A further 26 respondents selected the category ‘diverse’ when asked about their gender. No analyses were carried out for this gender category due to the small number of cases.

Twelve surveys were conducted between 2008 and 2025. In total, valid data on cannabis use in the past twelve months were available for 15,870 female and 16,624 male adolescents aged 12 to 17, as well as for 18,705 young women and 22,559 young men aged 18 to 25 (Table 1). Only 104 respondents had to be excluded from the analysis during this period due to a lack of data on cannabis use.

In 2025, 4.6 % of female and 7.2 % of male adolescents aged 12 to 17 reported having used cannabis in the past twelve months. Among women aged 18 to 25, this figure was 18.8 %, and among men of the same age, 31.6 %. Among female and male adolescents and young women, the 12-month prevalence of cannabis use in 2025 – that is, one year after partial legalisation – was slightly lower than in 2023. However, these differences were not statistically significant when the two years were compared directly (Table 1). 

Statistically significant differences compared to the reference year of 2025 were last observed among female adolescents in 2011 (2.8 %), among male adolescents in 2019 (10.8 %) and among young women in 2016 (11.4 %). Although there was a statistically significant increase among young men between 2023 and 2025, this was not a short-term development. Rather, it was a continuation of an increase that had already begun prior to partial legalisation.

**Figure 1:**
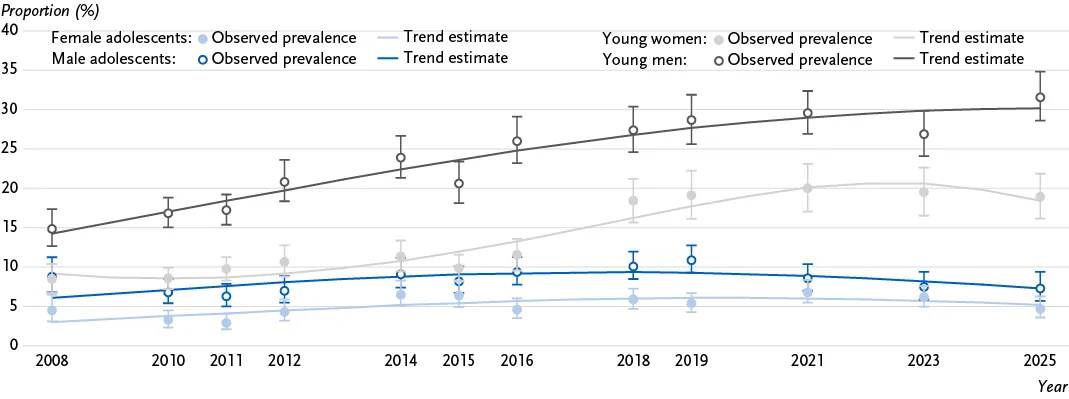
Trends in the 12-month prevalence of cannabis use among female and male adolescents from 2008 bis 2025^a^. Source: Drug Affinity Studies 2008 – 2025, Alcohol Surveys 2010 – 2021^b^ ^a^The figure shows the observed weighted prevalences (%) with 95 % confidence interval (95 % CI) and trend estimates ^b^Drug Affinity Studies were conducted in 2008, 2011, 2015, 2019, 2023 and 2025, and Alcohol Surveys in 2010, 2012, 2014, 2016, 2018, 2021

The estimated trends for 2008 to 2025 (Figure 1) followed a slightly inverted U-shaped pattern for both female and male adolescents, though this was somewhat more pronounced among males. Initially, the prevalence of cannabis use increased and then declined again. Among young women, the trend rose most sharply in the second half of the 2010s before levelling off significantly. The trend among young men rose steadily throughout the entire period from 2008 to 2025.

## 4. Discussion

One year after the partial legalisation of cannabis, no immediate, significant changes in the 12-month prevalence of cannabis use were observed among either female or male adolescents, or among young women, compared with 2023. In the longer term, a decline was observed among male adolescents compared with 2019. Although trend estimates suggest declines among female adolescents and young women, the effects are practically negligible. In a direct comparison of previous years with 2025, there were no significant differences among female adolescents since 2012 and among young women since 2018. In the group of young men aged between 18 and 25, the 12-month prevalence rose significantly from 2023 to 2025, which is part of a long-term upward trend observed since 2008. The Epidemiological Addiction Survey (ESA) reached similar conclusions, showing an increase in cannabis use among the 18- to 59-year-old population for the period from 2012 to 2021 and predicting a further rise regardless of any legislative changes [13]. In the 2024 wave of the ESA, which was conducted shortly after partial legalisation, the predicted further increase in consumption was indeed observed; however, the change was not statistically significant compared to 2021 [14].

Overall, the results suggest that the legislative changes introduced by the KCanG have had no short-term effect. This is consistent with the findings of the first report evaluating the law, which drew on other data sources from Germany in addition to the data presented here [15]. The studies mentioned at the outset from Canada and US states that had already legalised cannabis for recreational purposes reported, in some cases, significant increases in cannabis use among adults [3–6]. However, it should be emphasised that some of these studies were conducted some time after legalisation. In this respect, the results presented here do not yet allow for robust conclusions regarding the effects of the partial legalisation of cannabis in Germany. 

This requires further data collection at a greater interval following partial legalisation, as, for example, cannabis cultivation associations in Germany are still in the process of being established and the personal cultivation of cannabis plants is likely to continue to increase. Overall, the long-term monitoring of changes in cannabis use remains a key component in the initiation, monitoring and evaluation of prevention programmes. Prevention programmes continue to focus on adolescents and young adults. To raise their awareness of the health risks as early as possible and to empower them to use cannabis in a reflective and responsible manner, cannabis prevention measures have been re-established, intensified and continuously developed nationwide in recent years. An overview of the prevention programmes funded or supported by the Federal Institute for Public Health is presented in issue 3/2025 of the Journal of Health Monitoring [9].

## Figures and Tables

**Table 1:** 12-month prevalence of cannabis use among female and male adolescents and young adults from 2008 to 2025^a^. Source: Drug Affinity Studies 2008 – 2025, Alcohol Surveys 2010 – 2021^b^

**Year**	**Female**			**Male**		
	**Number of cases**	**Prevalence (%)**	**(95 % CI)**	**Number of cases**	**Prevalence (%)**	**(95 % CI)**
**12** **- to ** **17** **-year-old adolescents**						
2008	608	4.4	(3.0 – 6.4)	612	8.7	(6.7 – 11.2)
2010	1,280	3.2^*^	(2.2 – 4.4)	1,297	6.7	(5.3 – 8.4)
2011	997	2.8^*^	(2.0 – 4.0)	1,035	6.2	(4.9 – 7.8)
2012	1,029	4.2	(3.1 – 5.8)	996	6.9	(5.4 – 8.8)
2014	970	6.4	(5.0 – 8.2)	1,048	9.0	(7.3 – 11.1)
2015	1,583	6.3	(4.8 – 8.2)	1,710	8.1	(6.6 – 10.0)
2016	1,552	4.5	(3.4 – 5.9)	1,638	9.3	(7.7 – 11.2)
2018	1,536	5.8	(4.6 – 7.2)	1,646	10.0	(8.4 – 11.9)
2019	1,651	5.3	(4.2 – 6.6)	1,780	10.8^*^	(9.2 – 12.7)
2021	1,538	6.7	(5.4 – 8.3)	1,565	8.5	(6.9 – 10.3)
2023	1,638	6.1	(4.9 – 7.5)	1,761	7.4	(5.8 – 9.3)
2025	1,488	4.6	(3.5 – 6.2)	1,536	7.2	(5.6 – 9.3)
**18** **- to ** **25** **-year-old young adults**						
2008	903	8.3^*^	(6.6 – 10.3)	878	14.8^*^	(12.6 – 17.3)
2010	2,216	8.4^*^	(7.1 – 9.8)	2,198	16.8^*^	(15.0 – 18.8)
2011	1,480	9.6^*^	(8.2 – 11.2)	1,485	17.2^*^	(15.3 – 19.2)
2012	1,460	10.5^*^	(8.7 – 12.7)	1,505	20.8^*^	(18.3 – 23.6)
2014	1,361	11.2^*^	(9.4 – 13.3)	1,507	23.9^*^	(21.3 – 26.7)
2015	1,718	9.7^*^	(8.1 – 11.5)	1,981	20.6^*^	(18.1 – 23.4)
2016	1,658	11.4^*^	(9.5 – 13.5)	2,136	26.0^*^	(23.2 – 29.1)
2018	1,683	18.3	(15.6 – 21.2)	2,127	27.4^*^	(24.6 – 30.4)
2019	1,504	19.0	(16.1 – 22.2)	2,055	28.7	(25.6 – 31.9)
2021	1,580	19.9	(17.0 – 23.1)	2,306	29.6	(26.9 – 32.4)
2023	1,493	19.4	(16.5 – 22.6)	2,081	26.9^*^	(24.1 – 29.9)
2025	1,649	18.8	(16.1 – 21.8)	2,300	31.6	(28.6 – 34.9)

^a^The Figures show the unweighted number of cases and weighted prevalences (%) with 95 % confidence interval (95 % CI)

^b^Drug Affinity Studies were conducted in 2008, 2011, 2015, 2019, 2023 and 2025, and Alcohol Surveys in 2010, 2012, 2014, 2016, 2018, 2021

^*^Statistically significant difference from the reference value in 2025, with p < 0.05

## Footnotes

^1^ Until 13 February 2025: Federal Centre for Health Education, Germany.
